# Sex and Gender Differences in Physical Activity, Sedentary Behavior, and Sleep During Traumatic Brain Injury Recovery: A Narrative Review

**DOI:** 10.1002/ejsc.70248

**Published:** 2026-08-27

**Authors:** Goretti España‐Irla, Madeleine Perko, Emma M. Tinney, Mark C. Nwakamma, Martina Anto‐Ocrah, Timothy P. Morris

**Affiliations:** ^1^ Department of Physical Therapy, Movement, & Rehabilitation Sciences Northeastern University Boston Massachusetts USA; ^2^ Center for Cognitive & Brain Health Northeastern University Boston Massachusetts USA; ^3^ Department of Psychology Northeastern University Boston Massachusetts USA; ^4^ Division of General Internal Medicine Department of Medicine University of Pittsburgh Pittsburgh Pennsylvania USA; ^5^ Department of Epidemiology School of Public Health University of Pittsburgh Pittsburgh Pennsylvania USA; ^6^ Department of Applied Psychology Northeastern University Boston Massachusetts USA

**Keywords:** gender, physical activity, sedentary behavior, sex, traumatic brain injury

## Abstract

Traumatic brain injury (TBI) is a complex neurological condition with enduring consequences that extend well beyond the initial insult. Once regarded primarily as an acute event, TBI is now increasingly recognized as a chronic disorder associated with cognitive, emotional, and physical impairments. These sequelae contribute to diminished quality of life and elevated long‐term risks for adverse health outcomes. Despite growing awareness of its multifaceted nature, TBI recovery remains heterogeneous and is shaped by a range of individual‐level determinants that remain insufficiently understood. Among these determinants, biological sex and gender have emerged as critical yet under‐investigated influences on TBI outcomes. Sex refers to chromosomal, hormonal, and anatomical attributes, whereas gender encompasses socially constructed roles, identities, and norms that govern behavior, access to resources, and healthcare engagement. These dimensions interact in ways to influence not only TBI risk and presentation but also recovery trajectories. Although previous research has documented sex and gender disparities in TBI outcomes, limited attention has been given to how these factors intersect with lifestyle behaviors during rehabilitation. Modifiable lifestyle factors, including physical activity, sedentary behavior, and sleep, are essential for optimizing neuroplasticity, reducing inflammation, and promoting recovery following TBI. However, engagement in and physiological responses to these behaviors are shaped by both sex‐specific biological mechanisms and gendered sociocultural contexts. Recognizing and addressing these intersecting influences is crucial for advancing equitable and individualized rehabilitation strategies. This narrative review critically examines how physical activity, sedentary behavior, and sleep influence TBI recovery, with an emphasis on variation by sex and gender.

## Highlights

- Physical activity, sedentary behavior, and sleep are modifiable lifestyle factors with neurobiological relevance to traumatic brain injury (TBI) recovery, yet their interplay remains poorly characterized in clinical and rehabilitation research.
- Biological sex and gender are underexamined moderators of exercise‐related brain outcomes after TBI, with sex‐specific hormonal mechanisms (e.g., estrogen neuroprotection, testosterone‐driven tissue repair) and gendered sociocultural barriers shaping both engagement in and response to lifestyle‐based interventions.
- Advancing equitable, brain‐focused rehabilitation after TBI requires research frameworks that integrate sex‐ and gender‐disaggregated analyses, objective behavioral measures, and intersectional approaches to capture the full diversity of recovery experiences.

## 1 Introduction

Traumatic brain injury (TBI) is among the most prevalent and consequential neurological disorders globally, representing a substantial and escalating public health challenge. It affects individuals across all ages, sexes, and socioeconomic groups, and is now recognized not only as an acute event but as a chronic condition with enduring, often lifelong sequelae (Maas et al. 2022). It has been estimated that approximately half the world's population will sustain one or more TBIs over their lifetime (Maas et al. 2017). The consequences are far‐reaching, spanning cognitive, emotional, and physical domains, and are associated with reduced quality of life and elevated risks for neurodegenerative diseases (Crane et al. 2016; Huang et al. 2018; Li et al. 2017), cognitive decline (Moretti et al. 2012), stroke (Burke et al. 2013; Chen et al. 2011; Turner et al. 2021), psychiatric disorders (Howlett et al. 2022; Ponsford et al. 2018), and mortality (Harrison‐Felix et al. 2012; McMillan et al. 2014). These outcomes underscore the critical need for urgent, coordinated efforts in prevention, clinical management, long‐term care and inclusive research into the mechanisms, treatment, and recovery processes of TBI, particularly across diverse populations and individual‐level determinants of long‐term prognosis.

Among the determinants influencing TBI, biological sex and gender have emerged as crucial yet often underexamined variables. Sex refers to chromosomal, hormonal, and anatomical characteristics (Arboleda et al. 2014), whereas gender encompasses socially constructed roles, identities, and norms that shape behavior, experience, and access to care (Clayton and Tannenbaum 2017; World Health Organization 2015). These dimensions interact in complex ways, influencing TBI incidence, injury mechanisms, symptom expression, and recovery trajectories (Chaychi et al. 2022). Although several systematic reviews have explored sex and gender disparities in TBI outcomes (Chaychi et al. 2022; Gupte et al. 2019; Mollayeva et al. 2018; Mollayeva et al. 2021; Solomito et al. 2019), few have examined how these factors intersect with lifestyle behaviors. Modifiable lifestyle factors—such as physical activity, sedentary patterns, and sleep—play a critical role in post‐TBI recovery, yet they are deeply shaped by sex and gender through both biological and sociocultural pathways. Gender norms and social roles influence engagement in these behaviors, whereas biological sex may modulate their physiological effects on recovery (Mollayeva et al. 2018). Understanding these intersecting influences is essential for designing equitable, personalized rehabilitation strategies. Examining how sex and gender shape engagement in, and response to, lifestyle behaviors can guide more effective, tailored interventions to support long‐term resilience and well‐being following TBI.

Therefore, this narrative review aims to explore the impact of physical activity, sedentary behaviors, and sleep on recovery outcomes in individuals with TBI, with a focus on how these factors vary by sex and gender. By applying a sex‐ and gender‐informed lens, this review seeks to identify research gaps, highlight differential patterns in behavior and response to lifestyle‐based interventions, and inform the development of tailored, equitable rehabilitation strategies.

## 2 Methods

This narrative review draws on a structured but nonsystematic search of the literature conducted across PubMed, Scopus, and Web of Science. Search terms combined concepts relevant to traumatic brain injury (e.g., “TBI,” “mild traumatic brain injury,” “concussion”), sex and gender (e.g., “sex differences,” “gender differences”), and lifestyle behaviors (e.g., “physical activity,” “sedentary behavior,” “sleep”). No restriction was placed on publication date, allowing inclusion of both recent findings and earlier foundational or mechanistic studies relevant to sex‐ and gender‐related physiology. Reference lists of key articles were also screened to identify additional relevant sources. Eligible studies were peer‐reviewed, published in English, and involved human participants; animal or preclinical studies were excluded unless of direct mechanistic relevance. As a narrative review, this search strategy was not intended to be exhaustive or systematic, and no formal quality‐scoring or PRISMA‐based protocol was applied; rather, it was designed to provide broad, representative coverage of the literature relevant to the review's scope.

As this narrative review draws on a broad, nonsystematically screened literature base, we did not apply a formal evidence‐grading system (e.g., GRADE); instead, we indicate the nature and quality of the underlying evidence directly within the text, distinguishing meta‐analyses and systematic reviews from single cohort studies, animal models, or preliminary findings, and explicitly flagging results that remain contested or unreplicated.

## 3 Lifestyle Factors Influencing TBI Recovery

**FIGURE 1:**
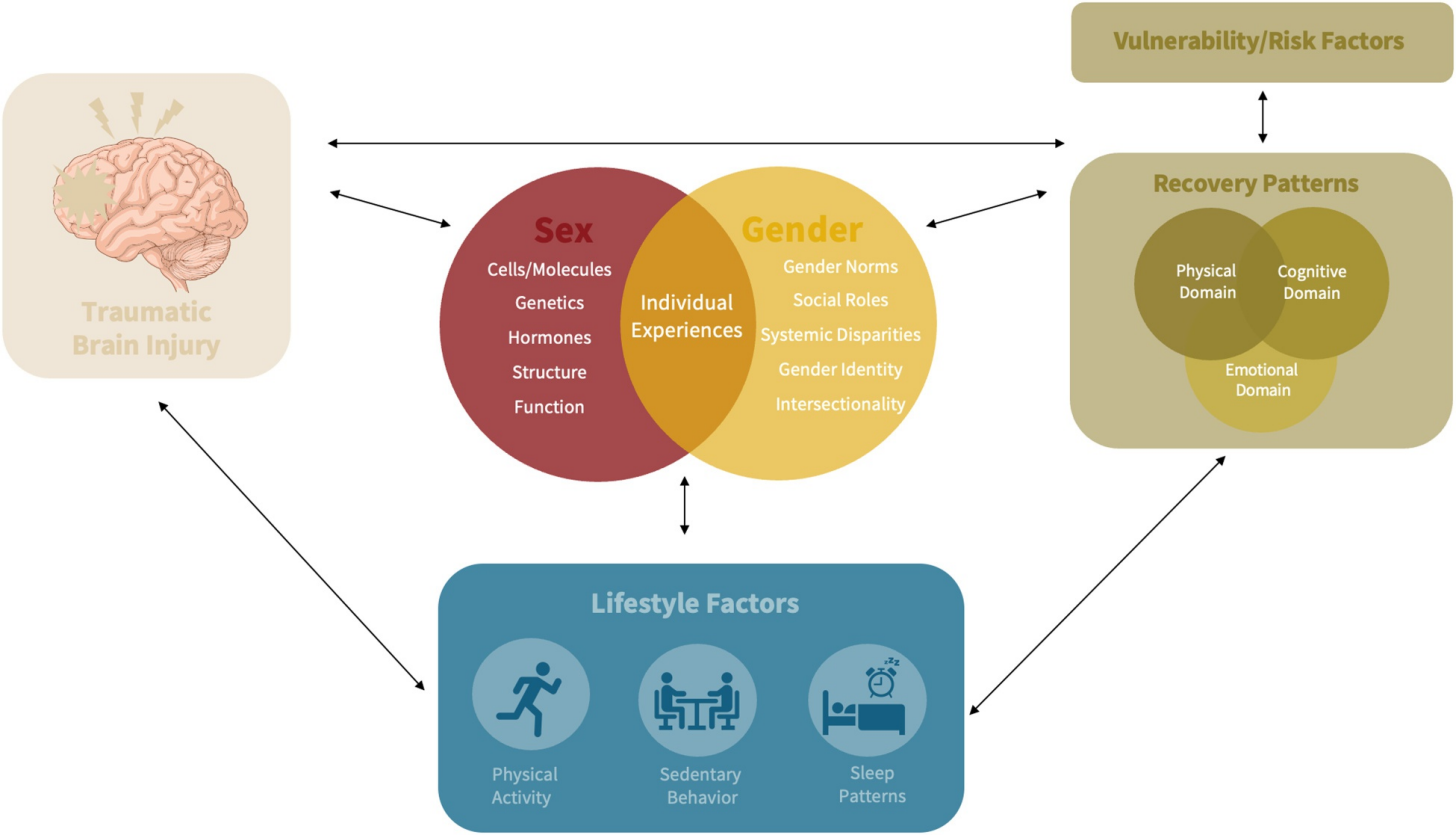
Interplay between traumatic brain injury, vulnerability, risk factors, and recovery patterns, with a focus on the influence of sex, gender, and lifestyle factors on outcomes.

Lifestyle factors likely play a critical role in shaping recovery outcomes following TBI yet are not currently considered in clinical practice. These factors, including, but not limited to, physical activity, sedentary behavior, and sleep are intricately linked to both biological processes and psychosocial aspects of rehabilitation. In the context of TBI, adopting and maintaining healthy lifestyle habits is not only essential for promoting neuroplasticity and brain health but also for supporting overall well‐being (Driver et al. 2019). However, the complex interaction between lifestyle choices and recovery is influenced by a variety of factors, including sex, gender, as well as socioeconomic status, and cultural background (Haarbauer‐Krupa et al. 2021). As a result, the influence of these factors on TBI recovery is not uniform, with evidence suggesting that men and women may experience and respond to lifestyle interventions differently (Figure 1). Understanding how these lifestyle factors interact within the context of TBI recovery is essential for developing personalized and effective interventions.

### 3.1 Exercise and Physical Activity

Extensive research in aging populations demonstrates that physical activity exerts beneficial effects on cognitive function and brain health, with both aerobic training and resistance training contributing to improved executive function, memory, and neuroplasticity (Bherer et al. 2013; Voss et al. 2013). Notably, emerging evidence highlights significant sex differences in both the efficacy of these interventions and the physiological mechanisms underlying their effects. Older females, despite generally engaging in less physical activity than males, have been shown to derive greater cognitive benefit from physical activity particularly in domains such as executive functioning (Barha and Liu‐Ambrose 2018; Colcombe and Kramer 2003). Meta‐analyses of randomized controlled trials have consistently reported larger effect sizes in studies with predominantly female samples, suggesting that biological sex may moderate the cognitive outcomes of exercise interventions (Barha et al. 2017). These sex‐based differences in physiological responses to exercise are shaped by anatomical, hormonal, and metabolic factors. Biological sex appears to moderate exercise efficacy, possibly due to differences in neurotrophic factor responses (e.g., BDNF, IGF‐1) and neuroplasticity mechanisms (Barha et al. 2019). Males and females exhibit distinct adaptations to physical activity, with variations in exercise capacity, muscular development, and cardiovascular function (Barha and Liu‐Ambrose 2018). These differences become more pronounced with age, particularly due to hormonal changes such as the decline in estrogen in postmenopausal females. Metabolic responses to exercise also vary by sex, which can impact the effectiveness of specific exercise approaches (Tarnopolsky 2008). Notably, these differences may, in part, be also related to sex‐specific variations in the effective dose of exercise, highlighting the need for further research to better understand and optimize exercise prescriptions across sexes.

Given the breadth of these sex‐specific differences in healthy populations, it is reasonable that similar divergences might exist in the context of mild TBI (mTBI). However, the influence of sex on the cognitive and physiological effects of physical activity and structured exercise interventions in TBI populations remains under‐investigated. Biological factors and sex hormones likely affect TBI‐outcomes differently. For example, estrogen has neuroprotective properties that promote neuroplasticity, reduce inflammation, and support neurogenesis (Bramlett and Dietrich 2001; Clevenger et al. 2018; Duncan 2020; Roof and Hall 2000). These effects may make females more responsive to aerobic exercise interventions, particularly during certain phases of the menstrual cycle or in hormone replacement therapy (Engler‐Chiurazzi et al. 2017). This raises the possibility that lifestyle interventions for females recovering from TBI could eventually be tailored to menstrual cycle phase. Injury occurring during the luteal phase, when progesterone is elevated and subsequently withdraws after injury, has been associated with poorer 1‐month outcomes in women with mTBI (Wunderle et al. 2014), a pattern supported by a recent scoping review (Carr and Fleddermann 2025). However, this remains a new and considerably understudied area, and any phase‐specific recommendations for TBI recovery or rehabilitation would be premature without further dedicated research. In animal models, such as ovariectomized rats, combining exercise with phytoestrogen treatments has been shown to improve bone density and osteoblast activity more effectively than exercise alone (Zhao et al. 2019). Rodent literature consistently reveals that estrogen loss leads to reduced physical activity (Clookey et al. 2019). However, direct studies on the interaction of exercise and estrogen in TBI are limited. Conversely, testosterone is linked to improved muscle strength, tissue repair, and resilience (Nam et al. 2018), suggesting that strength and resistance training could be particularly beneficial for male TBI survivors. Limited clinical trials indicate that combining testosterone therapy with exercise may optimize physical recovery (Ripley et al. 2020), but more research is needed to validate these findings (McLoughlin et al. 2022).

Menopause triggers significant changes in females physical health, primarily due to a decline in estrogen, which leads to reduced muscle mass and contributes to frailty. This sarcopenia is associated with cognitive decline, making females more vulnerable to physical and cognitive frailty, particularly after TBI (Sinder et al. 2024). Since menopause may heighten both the risk and severity of mTBI, understanding the complex relationship between menopause, frailty, and estrogen decline is crucial in studying TBI in postmenopausal females. Exercise has been identified as a promising intervention to manage the consequences of both menopause (Godoy‐Izquierdo et al. 2024; Marsh et al. 2023; McAndrew et al. 2009) and mTBI (Florez et al. 2024; Mercier et al. 2024; Vuu et al. 2022). Studies suggest that exercise can mitigate the negative outcomes of TBI, including cognitive decline, mood disorders, and postconcussion syndrome (Mercier et al. 2024; Zhang et al. 2022). However, menopausal females, especially those who are physically frail, may face challenges in adhering to exercise regimens due to factors such as preexisting conditions, fatigue, depression, and low motivation (Deng et al. 2023; Kalpakjian et al. 2024). Given the compounded effects of menopause‐related changes on physical and brain health, it is essential to consider these factors when evaluating the impact of mTBI and recovery in postmenopausal individuals (Sinder et al. 2024). Further research is needed to explore how exercise and other interventions can specifically address the unique needs of this population.

Although no studies to date have specifically examined sex differences in exercise responses following mTBI, research in poststroke populations provides some context. A systematic review investigating poststroke exercise interventions found no significant differences in executive function, memory, global cognition, or language outcomes when comparing studies with higher versus lower proportions of female participants (Khattab et al. 2021). However, limitations in current research, including variations in study designs and interventions, make it unclear whether sex should influence poststroke exercise prescriptions. This underscores the need for more clinical trials incorporating sex‐based analyses to adequately address this question.

Regarding comorbidities, registry‐based studies have shown that individuals recovering from TBI face an increased risk of cardiovascular (Eric Nyam et al. 2019; Hammond et al. 2019) and metabolic disorders (Kumar et al. 2018), as well as epilepsy (Ritter et al. 2016), stroke (Eric Nyam et al. 2019), and depression (Chan et al. 2017; Stein et al. 2019; Yeh et al. 2020), particularly in the chronic phase of recovery (Izzy et al. 2022). Obesity has been associated with higher symptomatology at 6 and 12 months following mTBI, as well as elevated concentrations of blood inflammatory markers throughout the recovery process (Eagle et al. 2023). These factors can exacerbate recovery challenges and may hinder the effectiveness of rehabilitation interventions. Research has consistently shown that obesity prevalence is higher in females than in males (Kranjac and Kranjac 2023), which can contribute to additional barriers to engaging in aerobic exercise (Baillot et al. 2021). Additionally, prior research revealed that female survivors of mTBI are at greater risk for developing posttraumatic stress disorder (PTSD) compared to males (Sudhakar et al. 2023), a condition that is linked to reduced physical activity, as well as higher rates of obesity and smoking (van den Berk‐Clark et al. 2018). Mental health conditions such as depression and anxiety are also more prevalent in females after TBI, especially in older women (van der Vlegel et al. 2022), further hindering their participation in rehabilitation programs and limiting the benefits of physical activity. Given the complex interplay between hormonal influences, sex differences, and comorbidities, it is crucial to design personalized physical activity interventions for TBI recovery that address these factors.

Gender also shapes physical activity engagement and rehabilitation outcomes after TBI, independent of the biological factors discussed above. Women, for instance, tend to report higher unmet needs related to physical activity and encounter more practical barriers, such as limited access to transportation, than men (Barton and Swinford 2021). These barriers often extend beyond access alone: gendered social roles can constrain women's own capacity to engage in physical activity during recovery, as women returning to work after TBI describe needing additional support with housekeeping and other domestic responsibilities, a “cumulative workload and double burden” that men less commonly report (Hanafy et al. 2022). Where research has stratified findings by gender, the picture that emerges is a modest one: at least one community‐based physical activity intervention found a slightly greater improvement in positive affect among women than men (Quilico et al. 2022). Yet such stratified findings remain the exception rather than the rule. A scoping review of community‐based physical activity interventions after moderate‐to‐severe TBI found that only 4 of 19 studies (21%) stratified results by sex or gender at all, and that the two terms were used interchangeably across nearly all studies, leaving any genuinely gender‐specific effects largely unexamined (Quilico et al. 2022).

Although sex and gender differences are acknowledged as potentially important factors in TBI exercise interventions and rehabilitation, current research fails to adequately address these biological and social determinants of TBI outcomes. Future studies must integrate gender‐sensitive approaches to improve the personalization and effectiveness of lifestyle‐based rehabilitation programs. By incorporating sex‐ and gender‐based analyses, researchers can develop interventions that address the unique needs of both men and women, ultimately enhancing recovery outcomes and ensuring more effective rehabilitation strategies for all individuals. Future research should explore how different types of exercise (e.g., aerobic vs. resistance training) influence both cognitive and physical recovery across sexes, also considering gender, hormonal status, age, and injury severity.

### 3.2 Sedentary Behaviors

Independent of physical activity, excessive sedentary behavior has been identified as a significant determinant of adverse health outcomes, with mounting evidence demonstrating its deleterious effects on cardiometabolic health and all‐cause mortality (Koster et al. 2012; Lavie et al. 2019; Matthews et al. 2012). Sedentary behavior, defined as time spent awake in a seated or reclined posture with minimal energy expenditure (Sedentary Behaviour Research Network 2012), has been robustly associated with a broad spectrum of negative health outcomes. These include heightened risk of depression (Teychenne et al. 2010) and anxiety (Teychenne et al. 2015), diminished emotional well‐being (Atkin et al. 2012; Endrighi et al. 2016), and an increased prevalence of sleep disorders (Kline et al. 2017; Yang et al. 2017). Additionally, sedentary behavior has been consistently linked to cognitive decline (Falck et al. 2017) and poorer overall physical health (Lavie et al. 2019). These findings underscore the critical role of sedentary behavior as a modifiable risk factor in the context of public health and clinical practice. In individuals with mTBI, sedentary behavior is a significant challenge, often exacerbated by fatigue, apathy, executive dysfunction, and reduced awareness following the injury. Physical activity levels decline markedly postinjury (Goverover et al. 2017), with increased sedentary time correlating with a reduced quality of life and heavier symptom burden (Driver et al. 2012; Mercier et al. 2021). Moreover, inactivity and irregular sleep patterns interact, creating a self‐reinforcing cycle where prolonged sitting disrupts the sleep drive, while poor sleep exacerbates fatigue and further reduces motivation for activity (Yang et al. 2017). Despite its importance, sedentary behavior in TBI has not been well documented, and the exploration of sex differences is even more limited.

In healthy populations, higher amounts of both sporadic and sustained moderate‐to‐vigorous physical activity (MVPA) have been associated with lower frailty in both sexes. Importantly, evidence shows that breaking up sedentary time with longer and more intense activity breaks is associated with reduced frailty in both sexes (Kehler et al. 2020). However, prolonged sedentary bouts have been more strongly linked to increased frailty in females than in males, suggesting a sex‐specific vulnerability to inactivity. In the context of TBI, where frailty can significantly worsen outcomes and hinder recovery, these findings underscore the importance of understanding how sedentary behavior, hormonal status, and sex intersect—particularly in postmenopausal females—who may experience compounding risks due to estrogen decline and limited physiological adaptation to physical activity. Tailored movement strategies that emphasize reducing sedentary time and promoting MVPA may be essential for improving recovery and resilience in this population.

Furthermore, emerging sex‐specific TBI‐related animal research suggests that males and females may exhibit distinct post‐TBI sedentary patterns influenced by differences in inflammation and sleep, with male rodents showing greater increases in sleep duration that may contribute to higher sedentary behavior (Saber et al. 2020). These findings highlight the need for further research, as human females generally report higher levels of sedentary behavior and significantly lower physical activity levels compared to males (Armstrong et al. 2018; Guthold et al. 2018), alongside poorer recovery outcomes following TBI. This suggests the presence of sex‐specific barriers and responses. However, more research is needed, particularly in the context of mTBI, to better understand how sex differences in sedentary behaviors influence recovery outcomes.

Beyond these sex‐based biological patterns, gender‐related sociocultural factors also shape sedentary behavior after TBI. Similarly, survivors of gender‐based violence (GBV) face unique challenges that may also influence their physical activity and sedentary behavior. GBV is a significant public health issue, involving violence directed at individuals due to their perceived gender or disproportionately affecting specific genders, such as women and members of the LGBTQI+ (Lesbian, Gay, Bisexual, Transgender, Queer/questioning, Intersex and other identities) community, due to unequal power dynamics (Hegarty et al. 2022). About one in three women will experience physical and/or sexual violence from a current or former intimate partner at least once in their lifetime (World Health Organization 2021). What is more, individuals who have suffered GBV are at an increased risk for mental health disorders, substance use, cardiovascular disease, and TBI (Baumann et al. 2019; Chandan et al. 2020; Dworkin 2020; Giordano et al. 2020; Haag et al. 2019; Pebole, Iverson, et al. 2024; Rees et al. 2014; Smith et al. 2022; Toccalino et al. 2023). A recent systematic review (Wheatley et al. 2024) investigating physical activity levels and sedentary behavior among individuals exposed to GBV revealed mixed findings, with most studies indicating lower physical activity levels among survivors, although these differences were not always statistically significant. Importantly, no study reported levels of sedentary behavior, a notable gap in the literature. This omission is concerning given that sedentary behavior is a known risk factor for all‐cause mortality and chronic conditions such as CVD, cancer, and diabetes, independent of physical activity levels (Patterson et al. 2018). Furthermore, individuals with mental health conditions, such as depression and PTSD, which are prevalent among GBV and TBI survivors, are known to engage in higher levels of sedentary behavior (Mason et al. 2019). This lack of gender‐based data limits our understanding of the broader health risks faced by GBV and IPV survivors and highlights the need for future studies to address this critical lifestyle behavior. Overall, survivors of GBV face unique barriers to participation in physical activity, including distrust, avoidance, fear of future trauma, unwanted attention and judgment, and low self‐esteem and self‐image (Darroch et al. 2024; Pebole, Singleton, et al. 2024). Individuals with histories of sexual violence and IPV survivors could benefit from specialized, gender‐sensitive approaches that prioritize their physical and psychological safety in exercise environments. These approaches may involve incorporating exercise into recovery plans for survivors, offering exercise groups and instructors of the same gender, providing education on the positive effects of exercise for those with experiences of GBV, and ensuring that bodily autonomy is emphasized in all exercise programs (Pebole et al. 2021; Pebole et al. 2024). In summary, there is a need for future research given the potential benefit of reducing sedentary behavior in the prevention and management of health consequences associated with experiencing GBV and TBI (Pebole et al. 2021). Higher‐quality research is needed to fill these gaps, especially studies that incorporate validated measures of physical activity and sedentary behavior, assess the recency of exposure to GBV and potential TBIs, and explore their connections to mental and physical health outcomes.

### 3.3 Sleep

In healthy populations, significant sex differences in sleep and circadian rhythms have been well documented. Females typically report poorer subjective sleep quality compared to males (Fatima et al. 2016; Lok et al. 2024; Vitiello et al. 2004). Additionally, females exhibit a higher prevalence of insomnia, restless legs syndrome, and parasomnias, whereas males are more commonly affected by obstructive sleep apnea (Lok et al. 2024). Circadian rhythms also differ by sex. Females tend to have earlier circadian phase markers and a shorter intrinsic circadian period than males (Lok et al. 2024). What is more, the female reproductive physiology in humans is under robust circadian control, with evidence showing that the timing and rhythmicity of key reproductive hormones vary across the menstrual cycle. For instance, during the follicular phase, multiple hormones, including estradiol, progesterone, follicle‐stimulating hormone (FSH), luteinizing hormone (LH), and sex hormone‐binding globulin (SHBG), exhibit clear circadian rhythms, whereas in the luteal phase, only FSH and SHBG maintain this rhythmicity (Rahman et al. 2019). Similarly, male reproductive physiology also follows circadian regulation, with testosterone secretion exhibiting a well‐characterized diurnal rhythm. Testosterone levels peak in the early morning around awakening and gradually decline throughout the day, reaching a nadir in the late afternoon (Gupta et al. 2000). These sex‐specific characteristics contribute to differential vulnerability to sleep and circadian disorders and may have broader implications for reproductive health. Another major life event that significantly impacts sleep patterns, highlighting sex‐specific differences, is the menopausal transition, during which sleep disturbances emerge as one of the most prevalent and debilitating symptoms experienced by females (Maki et al. 2024). However, despite these established differences, relatively little research has addressed how sex‐specific sleep and circadian patterns may influence outcomes in the context of TBI.

Sleep disturbances affect between 30% and 70% of individuals recovering from TBI, presenting as insomnia, hypersomnia, nightmares, and disrupted sleep architecture (Imbach et al. 2016; Ouellet et al. 2004). These issues arise from biochemical injury‐related changes and are worsened by cooccurring conditions like depression, anxiety, and pain (Fichtenberg et al. 2000; Ouellet and Morin 2006; Zeitzer et al. 2009). Poor sleep exacerbates cognitive deficits, fatigue, and recovery delays while reducing quality of life and increasing risk of neurobehavioral and mood disturbances (Bloomfield et al. 2010; Chaput et al. 2009; Fichtenberg et al. 2000; Kempf et al. 2010; Watson et al. 2007; Zeitzer et al. 2009). Sleep disruptions after TBI further hinder recovery by complicating pain management and reducing engagement in rehabilitation (Nabasny et al. 2020; Weber 2013; Worthington and Melia 2006). Emerging evidence points to structural brain changes associated with sleep disturbances following TBI. Axonal damage in regions like the anterior internal capsule has been linked to acute and long‐term sleep deficits, as well as cognitive impairments (Bajaj et al. 2017; Bottari et al. 2021; C.‐H. Cheng et al. 2020; Killgore et al. 2020; Lima Santos et al. 2022; Rojczyk et al. 2023; Tinney et al. 2024). However, evidence linking functional brain changes to sleep disruptions is more limited, with some studies indicating minimal effects (Killgore et al. 2020).

Sex and gender differences in sleep disturbances following TBI have also received limited attention, despite recent evidence suggesting that men and women may experience different patterns of sleep disruption and recovery (Ledger et al. 2020). Variations in commonly comorbid psychiatric disorders, such as posttraumatic stress disorder (PTSD), may provide an insight into how sex influences post‐TBI sleep‐related symptoms. The prevalence, consequences, and risk factors for PTSD differ between men and women, with women more likely to experience major depressive disorder, panic disorders, and suicide attempts, whereas men more frequently report alcohol abuse and dependence (Christiansen and Hansen 2015; Husky et al. 2018). Notably, the interplay of sex, gender, and age further complicates post‐TBI sleep disturbances. Evidence suggests that older females experience a greater impact of TBI on sleep disturbances compared to males (Ledger et al. 2020), emphasizing the role of age in moderating these effects. These differences underscore the need for tailored interventions that address both TBI‐related and comorbid conditions, such as PTSD and sleep disorders, by accounting for the distinct risk factors and coping mechanisms of each sex and gender. Just as it influences physical activity and sedentary behaviors, menopause also disrupts sleep quality, duration, and regulation, further distinguishing female sleep health from that of males. During this period, sleep disturbances become highly prevalent, often manifesting as frequent nighttime awakenings, reduced sleep quality, and insomnia (Baker et al. 2018; Bolge et al. 2010; Shaver and Woods 2015). These disruptions, driven by hormonal fluctuations, vasomotor symptoms, aging, and psychosocial stressors, significantly impact health, cognitive function, and overall well‐being (Baker et al. 2018). Although research has linked menopausal hormone therapy (MHT) and number of pregnancies to potential cognitive and brain health protection (Barth et al. 2024; de Lange et al. 2020), their role in mitigating post‐TBI sleep and cognitive decline remains largely unexplored. Given the established interplay between menopause, sleep, and brain health, future studies should investigate how menopausal status, age at onset, and MHT influence sleep‐related recovery trajectories in female TBI survivors.

Beyond these biological and hormonal patterns, gendered coping styles may also shape how post‐TBI sleep‐related distress is expressed. In a study exploring nightmares following TBI, as potential contributors to sleep disturbances, these were linked to increased substance abuse, negative affect, fatigue, maladaptive coping, depressive symptoms, and anxiety. Gender differences emerged, with women reporting more emotional distress, including negative affect and anxiety, whereas men with nightmares exhibited higher substance use. Additionally, women with nightmares showed more impulsivity than women without, a difference not observed in men (Nabasny et al. 2020). This pattern of women expressing distress emotionally, men expressing it behaviorally through substance use, mirrors a broader tendency for men to present psychological distress through externalizing behaviors rather than the emotional symptoms more commonly reported by women (Eggenberger et al. 2021), suggesting that gendered coping norms, rather than sleep pathology itself, may shape how post‐TBI distress is expressed. Beyond this, genuinely gender‐specific sociocultural data on sleep following TBI remain essentially absent from the literature; most existing research uses “gender” interchangeably with “sex,” reporting male–female comparisons rather than examining gender roles or broader sociocultural context. This represents an important gap for future research.

### 3.4 Sports‐Related TBI and Return to Play

Sport is one of the most common contexts in which TBI occurs, particularly among youth and young adults; and sex differences are evident from incidence through to recovery. A recent meta‐analysis of over 3 million youth athletes across 21 sports found an overall sport‐related concussion incidence of 1.41 per 1000 athlete exposures, with the highest rates in taekwondo, rugby union, and ice hockey (Ingram et al. 2025). Within sex‐comparable sports, incidence is not equally distributed: a meta‐analysis of 38 studies found that female athletes had significantly higher concussion rates than male athletes in soccer and basketball specifically, whereas no significant sex difference emerged in ice hockey, lacrosse, or swimming (J. Cheng et al. 2019). These differences extend into recovery: a 2026 systematic review of 41 studies and over 15,000 participants found that female patients consistently reported greater symptom burden, higher pain intensity, and longer recovery times for gait abnormalities and return to activity than males, alongside more extensive white matter alterations on neuroimaging, whereas males showed greater reductions in cerebral blood flow (Arachchi et al. 2026). Findings on return‐to‐play (RTP) timelines specifically, however, remain inconsistent: although some cohorts report that female athletes take significantly longer to begin an RTP progression (Stone et al. 2017), a large NCAA cohort study found that once an RTP progression was initiated, recovery trajectories converged and were largely similar between sexes, despite women reporting a greater symptom burden throughout (Caccese et al. 2024), suggesting that apparent sex differences in RTP timing may partly reflect differences in symptom reporting and access to care rather than differences in underlying physiological recovery. One study probed this reporting‐behavior explanation directly: rather than sex itself, conformity to risk‐taking norms predicted continued play while symptomatic, and only among female athletes, pointing to gender‐role conformity as a more precise explanatory variable than sex in at least this behavioral domain (Kroshus et al. 2017). A related, emerging area of sports science may eventually intersect with concussion rehabilitation: some researchers have proposed structuring resistance training around menstrual cycle phase, reporting superior strength and hypertrophy gains from follicular‐phase‐based training compared to luteal‐phase‐based training (Kissow et al. 2022). This conclusion remains contested, however; a subsequent umbrella review of the same evidence base concluded that current research is too methodologically inconsistent, particularly in verifying participants' actual cycle phase, to support any claim that the cycle phase meaningfully affects strength or hypertrophy outcomes (Colenso‐Semple et al. 2023). To date, this phase‐based training debate has developed independently of concussion research, and no study has yet examined whether structuring RTP exercise progression around menstrual cycle phase affects recovery outcomes, representing a genuinely open question at the intersection of these two research areas.

Beyond how symptoms are reported, the behaviors athletes engage in during recovery itself also shape outcomes. Physical activity, rather than strict rest, is now recognized as beneficial during concussion recovery. In a pediatric cohort, greater daily step counts and more frequent exercise sessions in the weeks following injury were associated with faster RTP clearance (Seehusen et al. 2021), consistent with current graduated RTP protocols that favor early, tolerable activity over prolonged rest. However, this relationship is not necessarily linear: in youth ice hockey players, higher moderate‐to‐vigorous physical activity in the first days after injury was instead associated with a longer time to medical clearance (Lishchynsky et al. 2019), suggesting that the timing and intensity of early activity, not simply its presence, may determine whether physical activity supports or delays recovery. Notably, sex differences in RTP appear concentrated earlier in the recovery process rather than during physical activity progression itself: in a cohort of 726 high school athletes, once cleared to begin the exercise portion of a stepwise RTP protocol, the time required to progress through each subsequent step was consistent across sex and age groups, even though female athletes had taken longer to reach that initial clearance point (Tamura et al. 2020). This suggests that once structured physical activity begins, sex‐based recovery differences described earlier in this section may narrow considerably.

Sedentary behavior, by contrast, has received less attention as a distinct construct in athletic populations, manifesting primarily as screen time, where sex differences emerge as an independent risk factor rather than a modifier of the screen‐time effect itself. A randomized clinical trial found that athletes who abstained from screen time for 48 hours after concussion recovered significantly faster than those permitted unrestricted use, with female sex independently predicting slower recovery in the same model (Macnow et al. 2021). A subsequent large cohort study similarly found that female sex predicted greater symptom severity over time, an effect larger than that of screen time itself (Cairncross et al. 2022).

Where physical activity and screen time show sex differences as risk factors layered onto behavior, sleep reveals something different: a genuine sex‐by‐behavior interaction. In a large cohort of adolescent athletes, female athletes recovered substantially more slowly than males overall (91 ± 95 vs. 58 ± 85 days), yet their recovery time was unaffected by how much sleep they reported. Male athletes, by contrast, recovered considerably faster with longer sleep duration (Hannah et al. 2021), suggesting sleep functions as a modifiable protective factor primarily for male athletes, whereas whatever drives female athletes' longer recovery lies elsewhere. Consistent with broader sex differences in symptom presentation, sleep disturbance itself was also more common in female than male collegiate athletes after concussion (42.0% vs. 29.3%) (Davis‐Hayes et al. 2017). Menstrual cycle phase may be a further, largely unexamined factor: in a small longitudinal study of elite female basketball players outside the concussion context, cycle phase itself showed only limited and inconsistent associations with sleep quality, whereas daily menstrual symptom burden was a more consistent predictor of poor sleep and recovery (Kullik et al. 2025), suggesting that if menstrual factors matter for sleep during TBI recovery, symptom burden rather than phase alone may be the more relevant variable to track. The physiological basis for these patterns remains largely unexamined: one home‐based monitoring study found reduced nocturnal cardiac activity in athletes returning to sport after concussion, correlating with drowsiness, but did not test for sex differences (Delling et al. 2023).

## 4 Gaps in Current Research and Future Directions

Although lifestyle factors like physical activity and sleep are increasingly linked to brain health after TBI, key questions remain about how, when, and for whom these interventions work best. Much of what we know stems from generalized findings, with limited attention to biological sex and gendered experiences, factors that may significantly shape recovery processes and treatment responsiveness. For example, although exercise is promoted as beneficial after mTBI, there is little agreement on the timing or intensity that is most effective. The brain's responsiveness to physical activity may shift depending on how soon it is introduced postinjury (Sánchez‐Martín et al. 2024), and this window may not be identical across individuals. Hormonal differences, age, and baseline health likely intersect with sex to influence neural plasticity and inflammation during recovery. What is therapeutic for one group could be neutral or even harmful for another. Emerging work suggests that MVPA can support neurobiological repair, but the optimal “dose” likely varies (Cabral et al. 2019), and sex‐specific physiology may play a role in this variance. Future studies should not only identify these windows of opportunity but also determine whether males, females, women and men—or people across the gender spectrum—experience different physiological and functional responses to the same intervention.

Similarly, sleep remains both under‐addressed and under‐theorized in TBI recovery research. Disturbed sleep is a common outcome of mTBI (Wickwire et al. 2016), yet its role in either supporting or hindering recovery remains unclear. Sleep architecture and circadian rhythms may be disrupted in distinct ways depending on sex, given known differences in hormonal regulation of sleep–wake cycles or biological life events such as menopause (Shaver and Woods 2015). Interventions that regulate sleep—whether behavioral, environmental, or pharmacological—could have variable outcomes depending on the individual's biological and social context. Additionally, research often treats physical activity, sedentary behavior, and sleep in isolation, even though these behaviors are part of a tightly interwoven twenty four‐hour cycle. There is growing interest in looking at behavior clusters rather than single habits. Interventions that combine activity, sleep hygiene, and mental well‐being strategies may be particularly impactful, but again, the interaction between these domains might not be uniform across sexes or gender identities. More nuanced models that consider social roles, caregiving burdens, or access to resources—factors often shaped by gender—are essential for building effective and inclusive interventions.

Beyond modifiable behaviors, it is equally important for mTBI research to investigate how these lifestyle factors intersect with nonmodifiable influences, apart from sex and gender, such as age, race, ethnicity, socioeconomic status, and cultural background. These intersecting dimensions shape disparities in recovery outcomes, research participation, and access to rehabilitation, and must be accounted for to develop equitable and effective care strategies.

Additionally, a key challenge across studies is methodological: overreliance on self‐report measures (Dick 2009), underrepresentation of diverse populations (D’Lauro et al. 2022; Giordano et al. 2020), and short follow‐up periods limit our understanding of long‐term outcomes and reduce the generalizability of findings. Methodologically, much of this imbalance stems from how studies measure sex and gender in the first place. Many rely on a single variable that conflates the two or infer gender from sex assigned at birth, making it impossible to identify genuinely gender‐based effects or to include nonbinary and transgender participants in analyses at all. Adopting standardized two‐step measures, collecting sex assigned at birth and current gender identity as distinct variables, would allow future TBI research to disentangle biological from sociocultural effects and to meaningfully include gender‐diverse populations, rather than defaulting to binary comparisons by necessity rather than design. Beyond identity categories alone, gradational femininity and masculinity scales could further enrich this approach by creating continuous and categorical measures of gender conformity, capturing a spectrum from lower to higher self‐perceived conformity and explicitly identifying nonconforming individuals, rather than treating gender as a fixed binary trait to be assigned rather than measured. Incorporating sex‐specific assessments, wearable technologies, advanced neuroimaging techniques, hormonal profiling, and well‐established behavioral assessments can offer more objective, biologically grounded insights into how the brain responds to lifestyle changes. These tools also allow for the examination of sex‐ and gender‐specific neural adaptations, which may otherwise go undetected.

Lastly, much remains unknown about why some individuals can engage in recovery‐promoting behaviors whereas others cannot. Factors like motivation, social support, and perceived safety can vary significantly between gender identities shaped by societal expectations, caregiving roles, or cultural norms. Programs that build and promote flexibility, foster community, and respect these contextual differences may boost adherence and broaden impact. Importantly, incorporating the voices of underrepresented groups, including women and gender‐diverse individuals, is critical to ensure that interventions reflect real‐world needs and preferences. There's growing recognition that sex and gender are not just demographic variables to control for, but key dimensions that influence how people experience injury, access care, and respond to treatment following mTBI (Chan et al. 2016; Fabricius et al. 2020; Mollayeva et al. 2018; Morgenroth and Ryan 2018; Solomito et al. 2019).

Although the studies reviewed here overwhelmingly rely on a binary sex/gender framework, evidence from outside the TBI literature suggests at least two distinct pathways by which gender‐diverse identity may shape physical activity, sedentary behavior, and sleep. The first is biological: a systematic review of gender‐affirming hormone therapy (GAHT) found that muscle strength increases during masculinizing GAHT and decreases during feminizing GAHT, whereas physical activity levels themselves remain largely unchanged, though the certainty of this evidence is limited by high risk of bias across studies (Nørlund et al. 2026). Consistent with this, transgender women on long‐term GAHT show cardiopulmonary capacity and muscle strength intermediate between cisgender women and cisgender men (Alvares et al. 2022). The second pathway is sociocultural rather than hormonal: gender minority stress, including stigma consciousness and everyday discrimination, is independently associated with worse sleep disturbance and shorter sleep duration in gender‐diverse adults (Caceres et al. 2022), and qualitative work identifies gender dysphoria, substance use, and unsafe or unaffirming environments as specific contributors to poor sleep health in transgender and nonbinary individuals (Harry‐Hernandez et al. 2020). A recent scoping review confirms these disparities are consistent across the broader sleep health literature on sexual and gender minority populations (Leonard et al. 2025). Given that this review has already established plausible hormonal mechanisms linking estrogen and testosterone to neuroplasticity, inflammation, and recovery after TBI, it is biologically and socioculturally plausible that both hormone‐related and minority‐stress‐related factors would similarly shape recovery trajectories for gender‐diverse individuals with TBI. Yet no studies to date have examined physical activity, sedentary behavior, or sleep specifically in gender‐diverse TBI populations, representing a critical and currently unaddressed gap. Future research must move beyond one‐size‐fits‐all models and toward approaches that reflect the biological and social complexity of TBI recovery (see Table 1 for a summary of identified research gaps and proposed directions).

**TABLE 1:** Summary of gaps in mTBI lifestyle research and corresponding recommendations.

Identified research gap	Proposed research direction
Insufficient understanding of the optimal timing and intensity of physical activity post‐mTBI across sexes and genders.	Identify sex‐ and gender‐specific windows for intervention and tailor exercise protocols to account for differences in injury recovery timelines and physiological responses.
Inadequate incorporation of sex and gender differences in response to sleep and exercise interventions.	Apply sex‐disaggregated analyses and integrate gender‐responsive frameworks throughout research design, implementation, and reporting.
Limited insight into how hormonal fluctuations impact mTBI recovery and lifestyles outcomes compounded by the lack of sex‐specific hormonal data in public open datasets.	Integrate hormonal profiling into study protocols and public datasets; examine the influence of biological life stages (e.g., puberty, menstrual cycle, pregnancy, menopause) on recovery patterns and modifiable lifestyles.
Under‐investigation of sex‐specific sleep disturbances and their implications for mTBI recovery.	Explore how biological sex and gender roles affect sleep architecture and recovery, and evaluate the effectiveness of tailored sleep interventions.
Fragmentation of lifestyle behaviors into isolated domains without considering sex/gender interactions.	Analyze physical activity, sleep, and sedentary behavior as interrelated constructs, and develop multimodal interventions that consider sex‐ and gender‐specific routines and needs.
Neglect of gendered social determinants such as caregiving burden, societal expectations, or gender norms in sleep, sedentary behavior and exercise intervention research.	Use qualitative and mixed methods approaches to uncover how gendered experiences and roles influence recovery, treatment adherence, and support needs.
Limited consideration of intersecting identities (e.g., race, socioeconomic status, culture) with sex and gender in shaping disparities in physical activity, sedentary behavior, and sleep outcomes after mTBI.	Use intersectionality‐informed frameworks to design studies that explicitly examine how overlapping identities shape lifestyle behavior patterns and recovery. Prioritize recruitment strategies that ensure representation across sex, gender, race, SES, and cultural backgrounds. Analyze how these factors interact to influence outcomes and tailor interventions accordingly.
Methodological limitations in lifestyles research including overreliance on self‐report, lack of objective measures, and short follow‐up, with inadequate attention to sex/gender differences.	Incorporate wearable technologies, neuroimaging, hormonal measures, and validated behavioral assessments to assess long‐term outcomes in physical activity, sedentary behavior, and sleep after mTBI. Specifically, investigate sex‐specific outcomes (e.g., differences in hormonal regulation) and gender‐specific outcomes (e.g., differential access to recovery resources, social roles, and behavioral norms influencing adherence and engagement).
Poorly understood differences in barriers to engaging in physical activity, reducing sedentary behavior, and maintaining healthy sleep across sex and gender identities.	Investigate how motivation, safety concerns, stigma, and access vary across sexes and gender identities; design flexible, inclusive lifestyle interventions to promote adherence.
Persistent underrepresentation of women and gender‐diverse individuals in mTBI research examining lifestyle behaviors.	Prioritize inclusive recruitment strategies and co‐design interventions with underrepresented populations to ensure relevance, safety, and equity.
Tendency to treat sex and gender as confounding variables rather than integral to understanding lifestyle behaviors and their role in recovery.	Reposition sex and gender as core biological and sociocultural dimensions that shape physical activity, sedentary behavior, and sleep patterns after mTBI, as well as responsiveness to lifestyle interventions.
Predominant reliance on a binary sex/gender framework in lifestyle research, which overlooks the experiences and physiological diversity of gender‐diverse individuals and those whose biological sex may not align with gender identity.	Expand research frameworks to recognize nonbinary, transgender, and intersex identities; collect gender identity and sex assigned at birth; investigate how medical transition (e.g., hormone therapy) and societal gender experiences influence physical activity, sedentary behavior, and sleep after mTBI; coproduce research with gender‐diverse participants to ensure relevance and inclusion.

## 5 Conclusion

Lifestyle behaviors are increasingly recognized as vital components of brain health, yet their role in mTBI recovery remains underexamined, particularly through the lens of sex and gender. Although it is clear that these behaviors can shape neural recovery, we still know little about how differences in biology and individual experiences influence who benefits, how much, and when. Physiological distinctions may interact with brain repair mechanisms such as neuroplasticity, inflammation, and sleep regulation, potentially altering the effects of physical activity and rest. Social realities also matter, unequal access to healthcare, caregiving demands, and gendered expectations can limit or shape how individuals engage with lifestyle interventions after injury. Moving forward, a more holistic, lifestyle‐centered approach, one that weaves together biological, psychological, and social dimensions, is essential to achieving meaningful, personalized recovery pathways after mTBI. By embedding sex and gender considerations into every stage of research and intervention, we can design strategies that not only improve outcomes but also promote equity in how recovery is understood, supported, and achieved.

## Author Contributions

**Goretti España‐Irla:** conception and design of the study, literature search, synthesis of findings, and drafting a significant portion of the manuscript. **Madeleine Perko:** literature search, synthesis of findings, critical review, and editing of the manuscript. **Emma M. Tinney:** critical review and editing of the manuscript. **Mark C. Nwakamma:** critical review and editing of the manuscript. **Martina Anto‐Ocrah:** critical review and editing of the manuscript. **Timothy P. Morris:** conception and design of the study, synthesis of findings, and drafting a significant portion of the manuscript.

## Funding

The authors have nothing to report.

## Ethics Statement

The authors have nothing to report.

## Consent

The authors have nothing to report.

## Conflicts of Interest

The authors declare no conflicts of interest.

## Data Availability

The data that support the findings of this study are available from the corresponding author upon reasonable request.
